# Association between adherence to MIND diet and general and abdominal obesity: a cross-sectional study

**DOI:** 10.1186/s12937-020-00531-1

**Published:** 2020-02-17

**Authors:** Azadeh Aminianfar, Ammar Hassanzadeh Keshteli, Ahmad Esmaillzadeh, Peyman Adibi

**Affiliations:** 1 Students’ scientific Research Center, Tehran University of Medical Sciences, Tehran, Iran; 2 Department of Community Nutrition, School of Nutritional Sciences and Dietetics, Tehran University of Medical Sciences, P.O. Box 14155-6117, Tehran, Iran; 3 Department of Medicine, University of Alberta, Edmonton, Alberta Canada; 4 Integrative Functional Gastroenterology Research Center, Isfahan University of Medical Sciences, Isfahan, Iran; 5 Obesity and Eating Habits Research Center, Endocrinology and Metabolism Molecular -Cellular Sciences Institute, Tehran University of Medical Sciences, Tehran, Iran; 6 Food Security Research Center, Department of Community Nutrition, Isfahan University of Medical Sciences, Isfahan, Iran

**Keywords:** MIND diet, General obesity, Central obesity, Body mass index, Cross-sectional

## Abstract

**Background:**

Recently, a new eating pattern called as “Mediterranean-DASH Intervention for Neurodegenerative Delay (MIND)” has been coined. Emerging studies are examining this dietary pattern with chronic conditions. We aimed to investigate the association between the MIND diet score and general and central obesity among adults.

**Methods:**

This cross-sectional study was conducted in a framework of the Study on the Epidemiology of Psychological Alimentary Health and Nutrition (SEPAHAN). Dietary information was collected using a validated self-administered 106-item Willett-format dish-based semi-quantitative food frequency questionnaire (DS-FFQ) in 6724 adults. Adherence to the MIND diet was examined based on components suggested in this eating pattern. Anthropometrics data were collected using a validated self-reported questionnaire. General obesity was defined as body mass index ≥30 kg/m^2^, and abdominal obesity as waist circumference > 102 cm for men and > 88 cm for women.

**Results:**

Mean age, BMI and WC in the study population was 36.8 ± 8.08 y, 24.9 ± 3.8 kg/m^2^ and 83.7 ± 16.02 cm, respectively. Overall, 9.5% of subjects were generally obese and 24.4 were abdominally obese. Examining the whole study population, we found no significant association between the MIND diet score and odds of general obesity, either before (ORs for comparing T3 vs. T1: 1.03; 95% CI: 0.83, 1.27; P-trend = 0.74) or after controlling for potential confounders (ORs for T3 vs. T1: 0.91; 95% CI: 0.67, 1.25; P-trend = 0.58). This was also the case for men and women when analyzed separately. We also failed to find any significant association between the MIND diet score and odds of abdominal obesity after controlling for potential confounders in the whole study population (ORs for T3 vs. T1: 1.00, 95% CI: 0.79, 1.27; P-trend = 0.87). However, women with the greatest adherence to the MIND diet were 19% less likely to be abdominally obese than those with the lowest adherence in crude model (OR = 0.81; 95% CIs: 0.67, 0.98; P-trend = 0.03). This association disappeared after controlling for potential confounders (OR = 0.87; 95% CIs: 0.66, 1.14; P-trend = 0.55).

**Conclusion:**

No significant association was observed between adherence to the MIND diet and odds of general and central obesity.

## Introduction

Obesity, a growing global health concern, has a dramatic increasing prevalence worldwide. It is defined as excess fat accumulation in the body and contributes to serious health problems [1, 2]. Prevalence of overweight and obesity is high, both in developed and developing countries. In Iran, the age-adjusted prevalence of overweight or obesity is estimated to be 42.8% in men and 57.0% in women [3].

Diet is a modifiable risk factor for obesity. Previous studies have investigated the associations between adherence to healthy dietary patterns including DASH diet (Dietary Approaches to Stop Hypertension) [4] as well as Mediterranean dietary pattern (MD) [5] and obesity, but the findings were inconsistent [6, 7]. Although, adherence to the MD diet was not associated with BMI in a large sample of European people, it was inversely associated with BMI and obesity in a Spanish population [8]. In addition, adherence to the DASH diet was inversely related to central obesity, but not to general obesity, among Iranian adult females [9]. In addition, a 4-month intervention with DASH diet had no significant effects on BMI among 124 overweight or obese Americans [10]. In patients with non-alcoholic fatty liver, lower weight and BMI was reported after 2 months of intervention with DASH diet [11]. Recently, the MIND diet (Mediterranean-DASH Intervention for Neurodegenerative Delay), which has been constructed based on brain-healthy and unhealthy foods, has been suggested [12]. It includes 15 components, of them 10 are brain-healthy foods (green leafy vegetables, other vegetables, berries, nuts, beans, whole grains, fish, poultry, olive oil and wine) and 5 are brain-unhealthy foods (cheese, butter or margarine, fast foods or fried foods, red meat and pastries or sweets). Few studies have investigated this dietary pattern in relation to chronic diseases. Most have found that high adherence to this eating pattern was associated with a slower cognitive decline, reduced risk of Alzheimer’s disease and lower odds of cognitive impairment [12–15]. Although the relationship between DASH and MD diets and obesity has separately been assessed [6, 7], no study is available linking the MIND diet to obesity. DASH and MD are both healthy dietary patterns. Each of these dietary patterns includes only some foods and food groups and no one includes all food items one might consume. For instance, DASH diet emphasizes on high consumption of fruits, vegetables, dairy, poultry and fish, nuts and lower intake of sodium and sweets [4]. It does not include the consumption of fast foods, full-fat dairies, margarines and butter. In addition, MD emphasizes on some foods included in the DASH diet, but it does not consider consumption of sweets and pastries [5]. Given that all foods consumed, even those that are not included in the scoring of DASH and MD, can affect human health, construction of new dietary scores that include all dietary components might help better predicting the risk of chronic disease. The main difference between the MIND diet and DASH and MD dietary patterns is that the MIND has been constructed based on foods that might be healthy or unhealthy to brain health. Given that the MIND diet includes fast foods, fried foods, butter, margarine, pastries and sweets, we hypothesized that this dietary pattern might help predicting obesity because all these foods are high-calorie foods that have been linked with obesity in earlier studies [16]. In addition, these foods were not included in the scoring method of DASH or MD diets.

In addition, obesity has a different and unique picture in Middle Eastern people [17, 18]. In a study based on data from 52 countries in 8 geographical regions, women in the Middle East had the highest rate of abdominal obesity than those in other regions of the world [18]. Moreover, people in the Middle East had the greatest mean BMI after those in US [18]. Immigrant women from the Middle East to European countries had higher prevalence of obesity and cardiovascular risk factors compared to women of European ethnicity [18]. Different obesity patterns and dietary habits in this region might provide some reasons for such a high prevalence [18, 19]. Consumption of high energy dense foods and refined carbohydrates including high amounts of rice and bread is among particular habits in the region [19]. Therefore, it seems that assessment of diet in relation to obesity in this part of the world is of great importance. Therefore, we aimed to investigate the relationship between adherence to the MIND diet and obesity in Iranian adults.

## Method

### Study design and population

The current cross-sectional study was carried out within the framework of Study on the Epidemiology of Psychological Alimentary Health and Nutrition (SEPAHAN), to evaluate the contribution of different lifestyle factors, including diet, to gastrointestinal disorders. Detail information about the study design, sample selection, data collection methods and characteristics of study participants have been reported previously [20]. The project was performed in Iranian adults working in 50 different health centers of Isfahan University of Medical Sciences in the central part of Iran. Using the sample size calculation formula for cross-sectional studies, considering the type I error of 0.05, obesity prevalence of 50% [3], and d = 0.05, we reached the minimum required sample size of 384 subjects. However, in SEPAHAN project, we had information for more than 5000 people. The whole project of SEPAHAN was approved by the Bioethics Committee of Isfahan University of Medical Sciences, Isfahan, Iran.

### Pre-study procedures

All participants provided written informed consent.

### Data collection throughout the study

Using a self-administered questionnaire, detail data on anthropometric measurements, socio-demographic characteristics and dietary intakes as well as data on physical activity was collected [20]. Eight thousand six hundred and ninety-one subjects completely responded to the sent questionnaire (response rate 86.16%). Because of the possibility of under- or over-reporting of dietary intakes, individuals with a total energy intake outside the range of 800 to 4200 kcal/day were excluded. These exclusions left 6724 and 5203 adults for the present analysis on general and abdominal obesity, respectively.

### Dietary assessment

Dietary data were collected using a self-administered 106-item Willett-format dish-based semi-quantitative food frequency questionnaire (DS-FFQ), which was designed for Iranian adults [21]. Detailed information about the design, foods included and the validity of the questionnaire was described elsewhere [22]. Common portion sizes were used to determine the amount of foods consumed. The questionnaire contained five main sections: (1) mixed dishes (cooked or canned, 29 items); (2) grains (different types of bread, cakes, biscuits and potato, 10 items); (3) dairy products (dairy, butter and cream, 9 items); (4) fruits and vegetables (22 items); and (5) miscellaneous food items and beverages (including sweets, fast foods, nuts, desserts and beverages, 36 items). Nine multiple-choice frequency response categories varying from “never or less than once a month” to “12 or more times per day” were designed for reporting dietary intakes of individuals. However, number of frequency response categories differed between common foods and infrequently consumed foods. For foods consumed infrequently, we omitted the high-frequency categories, whereas the number of multiple-choice categories increased for common foods with a high consumption. For instance, the frequency response for tuna consumption included six categories, as follows: never or less than once /mo, 1–3 times/mo, 1 times/wk., 2–4 times/wk., 5–6 times/wk. and 1–2 times/d, and for tea consumption, the frequency response included nine categories, as follows: never or less than 1 cup/mo, 1–3 cups/mo, 1–3 cups/wk., 4–6 cups/wk., 1 cup/d, 2–4 cups/d, 5–7 cups/d, 8–11 cups/d, and 12 or more cups/d. Food items were finally converted to grams per day. Daily intakes of nutrients were calculated using the US Department of Agriculture’s (USDA) national nutrient databank [23].

### Construction of the MIND diet score

Data derived from the FFQ was used for construction of the MIND diet score. Components of the MIND diet that we used in this study are presented in Table 1. In the original scoring method of the MIND diet [12], 15 dietary parameters were considered; of them 10 are brain-healthy food groups (green leafy vegetables, other vegetables, nuts, berries, beans, whole grains, fish, poultry, olive oil, and wine) and 5 are brain-unhealthy food groups (red meats, butter and stick margarine, cheese, pastries and sweets, and fast/ fried food). In the current study, olive oil and wine consumption was not included because of the lack of information in the original data set. To construct the MIND diet score, participants were classified based on tertile categories of intakes of these components. As scoring by tertiles would be least prone to misclassification, we used tertile categories of components instead of quantitative classifications. Individuals in the lowest tertile of brain-healthy food groups, including green leafy vegetables, other vegetables, nuts, berries, beans, whole grains, fish and poultry intake were given the score of 0, those in the middle tertile were given the score of 0.5 and those in the highest tertile were given the score of 1. Regarding brain-unhealthy food groups including red meats, butter and stick margarine, cheese, pastries and sweets, and fast/fried food intake, individuals in the lowest tertile were given the score of 1, those in the middle tertile were assigned the score of 0.5 and participants with the highest consumption of these food groups were given the score of 0. Finally, the overall the MIND diet score was calculated by summing up the scores of its components. Therefore, the overall the MIND diet score ranged from 0 and 13.

**Table 1:** MIND diet components

Brain healthy foods	
Green leafy vegetables	Cabbage, greens, lettuce
Other vegetables	Green/red peppers, raw carrot, potato, peas or lima beans, tomatoes, tomato sauce, eggplant, onion, cucumber
Berries	Strawberries (strawberry, cherries, fresh berries)
Nuts	Walnuts, pistachios, hazelnuts, almonds, peanuts
Whole grains	Dark bread (Iranian)
Fish	Fish
Beans	Beans, lentils, peas, chick pea, mung bean
Poultry	Chicken
Brain unhealthy foods	
Butter, margarine	Butter, margarine, animal fats
Cheese	Cheese
Red meat and products	Red meat, hamburger, sausages
Fast fried foods	French fries, pizza
Pastries and sweets	Biscuit, cake, chocolate, ice cream, confections, cocoa, Gaz (an Iranian confectionery made of sugar, nuts and tamarisk), Gooshfil (an Iranian confectionery made of white flour and sugar)

### Anthropometric assessment

Data on height, weight and waist circumference (WC) were collected using a self-reported questionnaire. The validity of self-reported anthropometric values in these participants has been reported in our previous study [24]. The correlation coefficients for the self-reported weight, height and WC versus measured values were 0.95 (*P* < 0.001), 0.83 (*P* < 0.001) and 0.60 (*P* < 0.001), respectively. Body mass index (BMI) was calculated as weight (kg) divided by height (m) squared. Obesity was defined as having BMI ≥30 kg/m^2^. Abdominal obesity was defined based on WC and NCEP-ATP III criteria [25]. Participants were categorized into two groups based on their WC: normal (≤88 cm for women and ≤ 102 cm for men) and abdominally obese (> 88 cm for women and > 102 cm for men).

### Assessment of other variables

Self-administered questionnaires were used to collect information on age, gender, marital status (single/married), education (high school diploma or below/above high school diploma), smoking status (non-smoker/former smoker/current smoker), family size (≤4/> 4 members) as well as home ownership (owner/non-owner). Physical activity levels of participants were assessed using the General Practice Physical Activity Questionnaire (GPPAQ), a simple and four-level physical activity index (PAI) reflecting an individual’s current physical activity [26]. Participants were classified into four categories: active (> 3 h/ week), moderately active (1–3 h/week), moderately inactive (< 1 h/week), and inactive (no physical activity). In the current analysis, we classified participants into two categories: < 1 h/week (active and moderately active) or ≥ 1 h/week (moderately active and inactive).

### Statistical analysis

General characteristics of subjects across tertiles of the MIND diet score were expressed as means ± SDs for continuous variables and percentages for categorical variables. To examine the differences across tertiles, we used ANOVA for continuous variables and a chi-square test for categorical variables. Dietary intakes of study participants across tertiles of the MIND score were compared using ANCOVA, adjusted for age, sex and energy intake, except for dietary energy intake, which was only adjusted for age and sex. We used binary logistic regressions to estimate ORs and 95% CIs for the presence of general and abdominal obesity across tertiles of the MIND score in crude and multivariable-adjusted models. Age and total energy intake were controlled for in the first model. Further adjustments were made for marital status (married, single, divorced and widowed), physical activity (< 1 h/week/≥1 h/week), smoking (non-smoker, former smokers and current smokers), family size (≤4/> 4 members), breakfast skipping (skippers/non-skippers), educational levels (university graduate and below that), home ownership (owner/non-owner), fruit (other than berries), refined grains, dairy (other than cheese and ice-cream) and total fat intake in the second model. Since the association between the MIND diet and obesity might be affected by the other obesity-related food groups, that are not included in the MIND diet, we adjusted the analyses for these food groups including: fruit (other than berries that are included in the MIND diet), refined grains, dairy (other than cheese and ice-cream) and total fat intake. P for trend was determined by considering tertiles of the MIND score as linear continuous variables in the logistic regression analysis. All statistical analyses were done using the Statistical Package for Social Sciences (version 20; SPSS Inc.). *P* < 0.05 was considered statistically significant.

## Results

Mean age of study participants was 36.8 ± 8.08 (men: 39.4 ± 8.3 and women: 35.3 ± 7.5). Mean BMI and WC in the whole study population was 24.9 ± 3.8 kg/m^2^ and 83.7 ± 16.02 cm. Overall, 9.5% of study participants were generally obese (men: 9.2 and women: 9.7%) and 24.4% had abdominal obesity (men: 11.2 and women: 32.7%). Compared with those in the bottom tertile, participants in the top tertile of the MIND diet score had higher BMI, were older, more likely to be female, university graduated, physically active and more likely to skip their breakfast and less likely to be house owner (Table 2).

**Table 2:** Baselines characteristics of participants according to tertiles of MIND diet score (*n* = 6724)

Variables	Tertiles of MIND diet score			***P***-value^**b**^
	1 (0.5- < 6)	2 (6–7)	3 (> 7–11.5)	
Age, y^a^	35.9 ± 7.7	37.0 ± 8.1	37.7 ± 8.2	< 0.001
Gender (female) (%)	56.5	60.3	64.4	< 0.001
Married (%)	82.4	83.8	82.3	0.80
University graduated (%)	57.2	58.9	65.3	< 0.001
Family size (> 4 people) (%)	10.1	10.7	9.7	0.51
Current smoker (%)	5.4	4.6	4.2	0.30
Physically active (≥1 h/week) (%)	29.0	32.6	40.0	< 0.001
Breakfast skipping (≥4 times/week) (%)	74.2	75.9	80.3	< 0.001
Home ownership (non-owner) (%)	33.6	31.9	29.4	0.02
BMI (kg/m^2^)	24.8	24.9	25.1	0.03
Waist circumferences (cm)	83.8	83.7	83.7	0.99
Obesity (%)^c^	9.1	10.2	9.4	0.41
Abdominal obesity (%)^d^	24.1	24.8	24.3	0.89

^a^ Data are mean ± standard deviation (SD)

^b^ Obtained from ANOVA or chi-square test, where appropriate

^c^ BMI ≥ 30 kg/m^2^

^d^ Having waist circumference > 102 cm for men and > 88 cm for women

Dietary intakes of study participants across tertiles of the MIND score are provided in Table 3. Participants in the third tertile had lower intakes of red meat, grains, fat and vitamin B12 and higher intakes of fruits, vegetables, organ meat, white meat, legumes, nuts, as well as energy, carbohydrates, proteins, fiber, folate, vitamin B6 and magnesium compared with those in the first tertile.

**Table 3:** Dietary intakes of study participants across tertiles of MIND diet score^a^ (*n* = 6724)

Variables	Tertiles of MIND diet score			***P***-value^**b**^
	1 (0.5- < 6)	2 (6–7)	3 (> 7–11.5)	
Food groups (g/d)				
Fruits	206 ± 4.6	272 ± 4.2	364 ± 4.8	< 0.001
Vegetables	171 ± 2.5	208 ± 2.3	262 ± 2.6	< 0.001
Dairy products	336 ± 6.0	326 ± 5.6	336 ± 6.35	0.32
Red meat	75.0 ± 0.82	69.5 ± 0.76	60.7 ± 0.85	< 0.001
Organ meat	2.37 ± 0.14	2.89 ± 0.13	2.99 ± 0.15	0.006
White meat	79.09 ± 1.0	87.1 ± 0.99	98.4 ± 1.13	< 0.001
Legumes	37.6 ± 0.79	45.7 ± 0.73	50.6 ± 0.82	< 0.001
Nuts	5.7 ± 0.27	8.23 ± 0.25	11.1 ± 0.28	< 0.001
Grains	404 ± 3.61	385 ± 3.35	363 ± 3.7	< 0.001
Sweets	23.4 ± 0.60	23.8 ± 0.55	22.0 ± 0.63	0.08
Nutrients				
Energy, kcal/d	2156 ± 18.3	2388 ± 17.2	2588 ± 19.2	< 0.001
Carbohydrate, g/d	284 ± 1.11	290 ± 1.02	298 ± 1.16	< 0.001
Protein, g/d	86.82 ± 0.33	88.0 ± 0.31	90.5 ± 0.35	< 0.001
Fat, g/d	101 ± 0.41	99.5 ± 0.38	95.9 ± 0.43	< 0.001
Fiber, g/d	19.9 ± 0.12	22.5 ± 0.11	25.6 ± 0.12	< 0.001
Folate, μg/d	545 ± 2.7	564 ± 2.54	585 ± 2.8	< 0.001
Vitamin B6, mg/d	1.92 ± 0.009	2.00 ± 0.009	2.09 ± 0.009	< 0.001
Vitamin B12, μg/d	3.15 ± 0.02	2.97 ± 0.02	2.53 ± 0.02	< 0.001
Magnesium, mg/d	302 ± 1.16	326 ± 1.08	358 ± 1.22	< 0.001

^a^Data are Mean ± standard error (SE)

^b^All values were adjusted for age, sex and energy, except for dietary energy intake, which was only adjusted for age and sex using ANCOVA

Crude and multivariable-adjusted ORs (95% CIs) for general obesity across tertiles of the MIND diet score are indicated in Table 4. When examined in the whole study population, no significant association was observed between the MIND diet score and general obesity, either before [OR for T3 vs. T1: 1.03 (95% CI: 0.83, 1.27), P-trend = 0.74] or after controlling for potential confounders [OR for T3 vs. T1: 0.91 (95% CI: 0.67, 1.25), P-trend = 0.58]. This was also the case for men and women, separately. After adjustment for potential confounders, men in the highest tertile of the MIND score, compared with those in the lowest tertile, had a 4% non-significant greater odds of general obesity (95% CI: 0.63, 1.71; P-trend = 0.85). However, among women, those with the greatest adherence to the MIND diet were 16% less likely to be generally obese compared with those with the lowest adherence (95% CI: 0.56, 1.27; P-trend = 0.43), albeit non-significantly.

**Table 4:** Odds ratio (95% CI) for general obesity according to tertiles of MIND diet score and stratified by gender

Variables	Tertiles of MIND diet score			P-trend
	1 (0.5- < 6)	2 (6–7)	3 (> 7–11.5)	
Whole population				
Subjects, n	2136	2415	2173	
Crude	1.00	1.13 (0.93, 1.37)	1.03 (0.83, 1.27)	0.74
Model I^a^	1.00	1.003 (0.81, 1.24)	0.90 (0.71, 1.14)	0.40
Model II	1.00	0.98 (0.74, 1.29)	0.91 (0.67, 1.25)	0.58
Men				
Crude	1.00	1.17 (0.85, 1.60)	1.20 (0.85, 1.68)	0.27
Model I^b^	1.00	1.17 (0.83, 1.65)	1.22 (0.83, 1.79)	0.28
Model II	1.00	1.10 (0.72, 1.68)	1.04 (0.63, 1.71)	0.85
Women				
Crude	1.00	1.06 (0.82, 1.37)	0.95 (0.72, 1.24)	0.71
Model I^b^	1.00	0.91 (0.70, 1.20)	0.77 (0.57, 1.03)	0.07
Model II	1.00	0.93 (0.64, 1.34)	0.84 (0.56, 1.27)	0.43

Data are OR (95% CI)

Model I^a^: adjusted for age, sex and energy intake

Model I^b^: adjusted for age and energy intake

Model II: additionally, adjusted for marital status, education, family size, smoking status, physical activity, breakfast skipping, home ownership, fruit (other than berries), refined grains, dairy (other than cheese and ice-cream) and total fat intake

Table 5 provides crude and multivariable-adjusted ORs (95% CIs) for abdominal obesity across tertiles of the MIND diet score. No significant association was seen between adherence to the MIND diet and abdominal obesity in the whole population, either before [OR for T3 vs. T1: 1.0 (95% CI: 0.85, 1.18), P-trend = 0.90] or after controlling for potential confounders [OR for T3 vs. T1: 1.0 (95% CI: 0.79, 1.27), P-trend = 0.87]. Among men, participants in the highest tertile of the MIND diet score had a 47% non-significant greater odds of abdominal obesity (95% CI: 0.90, 2.38; P-trend = 0.37). However, women with the greatest adherence to the MIND diet had 19% significant lower odds of abdominal obesity (95% CI: 0.67, 0.98; P-trend = 0.03) in the crude model. There was no significant association between the MIND diet score and abdominal obesity after controlling for potential confounders in women (OR for T3 vs. T1: 0.87; 95% CI: 0.66, 1.14; P-trend = 0.55).

**Table 5:** Odds ratio (95% CI) for abdominal obesity according to tertiles of MIND diet score and stratified by gender

Variables	Tertiles of MIND diet score			P-trend
	1 (0.5- < 6)	2 (6–7)	3 (> 7–11.5)	
Whole population				
Subjects, n	1755	1937	1527	
Crude	1.00	1.03 (0.89, 1.20)	1.0 (0.85, 1.18)	0.90
Model I^a^	1.00	1.0 (0.85, 1.17)	0.93 (0.79, 1.11)	0.46
Model II	1.00	0.95 (0.77, 1.18)	1.0 (0.79, 1.27)	0.87
Men				
Crude	1.00	1.04 (0.74, 1.45)	1.35 (0.95, 1.92)	0.09
Model I^b^	1.00	1.05 (0.97, 1.99)	1.39 (0.97, 1.99)	0.07
Model II	1.00	1.05 (0.68, 1.62)	1.47 (0.90, 2.38)	0.37
Women				
Crude	1.00	0.96 (0.97, 1.15)	0.81 (0.67, 0.98)	0.03
Model I^b^	1.00	0.98 (0.82, 1.18)	0.83 (0.69, 1.01)	0.07
Model II	1.00	0.94 (0.71, 1.17)	0.87 (0.66, 1.14)	0.55

Data are OR (95% CI)

Model I^a^: adjusted for age, sex and energy intake

Model I^b^: adjusted for age and energy intake

Model II: additionally, adjusted for marital status, education, family size, smoking status, physical activity, breakfast skipping, home ownership, fruit (other than berries), refined grains, dairy (other than cheese and ice-cream) and total fat intake

## Discussion

In this cross-sectional study, we found no significant association between adherence to the MIND diet and general obesity in Iranian adults. Despite a significant inverse association between the MIND diet and abdominal obesity among women in crude model, the association became non-significant after adjustment for potential confounding factors. To the best of our knowledge, this is the first study examining the relationship between the MIND diet score and general obesity and central adiposity in adult population.

Overweight and obesity are associated with increased risk of morbidity and mortality [27]. Their prevalence is dramatically increasing in recent years [28, 29]. Despite the knowledge on several dietary patterns to prevent chronic conditions, it seems that such eating recommendations were unable in controlling obesity. Therefore, developing new effective dietary approaches might be required. Recently, the MIND diet has been suggested as a new healthy dietary pattern that is inversely associated with risk of Alzheimer disease and cognitive decline [12, 13]. Although this dietary pattern has been constructed by combining Mediterranean and DASH dietary patterns, it has some advantages over these eating patterns. The MIND diet uniquely emphasizes on the consumption of berries and beans, which might influence body weight because of their content of resveratrol and fiber [30, 31]. In addition, low intake of calorie-dense foods, including fast and fried foods, pastries and sweets and butter and margarines, in this dietary pattern might further explain its probable beneficial effect on weight control [32–34].

In the current study, we observed no significant association between adherence to the MIND diet and odds of general and abdominal obesity. As mentioned before, the MIND diet is derived from the combination of DASH and MD diet. Although there is no study examining the linkage between the MIND diet and obesity, the relationships between DASH and MD diet and obesity have been investigated previously. In agreement with our findings, some studies have shown no significant association between DASH [10, 35, 36] or MD diet [37, 38] and obesity. In a clinical trial by Smith et al., a 4-month intervention of DASH diet had no significant effects on weight and BMI in 124 overweight or obese (BMI > 25 kg/m^2^) participants [10]. Also, in the European Prospective Investigation into Cancer and Nutrition (EPIC) Study among a large population of 23,597 men and women, adherence to MD diet was not associated with BMI [38]. However, some other investigations reported an inverse association between adherence to DASH and MD diet and BMI [6–8, 11, 39] and WC [40, 41]. In a recent meta-analysis on 13 randomized clinical trials (10 for body weight and BMI and 2 for WC), consumption of DASH diet resulted in a decreased weight, BMI and WC. The effect was greater in overweight/obese people [6]. Moreover, five-point increase in MD diet score was associated with a mean decline of 1.54 cm in sex-, age- and height-adjusted WC in another study [41]. The disagreements between our findings and those for DASH and MD might be explained by the different fiber content of MIND, DASH and MD diets. DASH and MD contain high amounts of dietary fiber than MIND diet because they are rich in fruits, while MIND diet does not contain all fruits included in the DASH and MD diets. In addition, although MIND diet includes beans, it must be kept in mind that due to nutritional transition consumption of beans in the framework of traditional diets in Asian countries has reduced [42]. Therefore, despite the consideration of beans consumption in the MIND diet, the whole beans intake in the diet was not so high. Another difference between the MIND diet and the other two dietary patterns (DASH and MD) is dairy consumption. Earlier studies have shown that greater dairy intake might be associated with a lower risk of obesity [43, 44]. This has even been shown in a meta-analysis that weight-loss diets containing high dairy might decrease body weight and fat much more than the diets with a low dairy content [45]. In the MIND diet, only cheese consumption was included while in the DASH and MD diets, consumption of all dairy products is encouraged.

We found non-significant decreased odds of both general and abdominal obesity in women in the top tertile of MIND diet score, compared with those in the bottom tertile. This non-significant association was positive for both general and abdominal obesity in men. In other words, men in the third tertile of MIND diet score had non-significant greater odds of general and abdominal obesity compared to those in the lowest tertile. Given the large sample size and sufficient study power in the study, these findings were not significant. Therefore, they do not seem to be of importance. However, one possible explanation for these findings might be sex differences in the obesity pattern and fat distribution. Estrogen in women enforces fats to accumulate in hip, while lack of estrogen in men results in enlarged waist circumference. It must also be kept in mind that women in the present study were young persons with a mean age of almost 35. Most women at this age category are sensitive about their body shape. This results in adopting a healthy lifestyle in this age category, which can in turn lead to a lower prevalence of obesity than men.

Generally, MIND diet, which was basically developed based on brain-healthy and –unhealthy foods [12, 13, 46], seems to have some limitations to predict other chronic conditions including obesity. Based on the findings of the current study, MIND diet per se cannot predict obesity and abdominal obesity. However, if fruits other than berries and dairy products other than cheese are included in this dietary pattern, it might be a healthy eating pattern to prevent obesity.

This study had several strengths. To the best of our knowledge, this is the first study investigating the linkage between MIND diet score and general and central obesity. The analysis was performed on a large sample of adults in a Middle Eastern country, where the information on diet-disease associations are limited. In addition, we controlled for several potential confounders. Dietary intakes were assessed by a validated dish-based questionnaire which provides accurate and reliable information. Nevertheless, some limitations need to be considered when interpreting our findings. Due to the cross-sectional design of our study, the causality cannot be inferred. Furthermore, individuals with high body fat and weight may have altered their diets in an effort to control their obesity status. In addition, although we controlled the analysis for several potential confounders, residual confounding cannot be excluded. Moreover, some degree of measurement error and misclassification must be noted due to the use of FFQ. In addition, due to lack of any specific Iranian food composition table, we used USDA nutrient databank to compute daily intakes of nutrients. Since participants in the study were adults working in 50 different health centers across Isfahan province; therefore, generalization of our findings to the general Iranian population must be done with caution. It must also be taken into account that obesity and abdominal obesity were defined based on self-reported anthropometric variables. Although, our validation study revealed that these data provide reasonably valid information, others had suggested the use of accurate methods to examine body composition. Dual X-ray absorptiometry (DXA) and bioelectrical impedance device (BIA) are accurate methods for measuring body composition. Although some studies have shown their usefulness to examine body composition in healthy or unhealthy individuals [47, 48], some investigations reported that the application of BIA might overestimate visceral adipose tissue in overweight or obese subjects [47]. In addition, we defined central obesity based on WC in the current study. Earlier studies have shown that the application of DXA can accurately show fat mass and visceral adipose tissue, albeit not in normal-weight individuals [49, 50].

## Conclusion

In conclusion, no significant associations were found between adherence to MIND diet and odds of general and central obesity in this cross-sectional study among adults. This finding suggests that despite the usefulness of MIND diet for having a healthy brain; it might not be able to predict other chronic conditions like obesity because of low content of fruits and dairy. Given the limitations we had in the study, further investigations are needed to further examine the association between this dietary pattern and risk of obesity.

## Data Availability

The datasets used and analyzed during the current study are available from the corresponding author on reasonable request.

## Abbreviations

BMI: Body mass index
DASH: Dietary Approaches to Stop Hypertension
DS-FFQ: Dish-based semi-quantitative food frequency questionnaire
GPPAQ: General Practice Physical Activity Questionnaire
MD: Mediterranean dietary pattern
MIND: Mediterranean-DASH Intervention for Neurodegenerative Delay
PAI: Physical activity index
SEPAHAN: Study on the Epidemiology of Psychological Alimentary Health and Nutrition
USDA: United state department of agriculture
WC: Waist circumference
